# Phytoplankton With Flexible Pigment Content Disadvantaged by Projected Future Decrease in Variability of the Ocean Light Spectrum

**DOI:** 10.1111/gcb.70671

**Published:** 2026-01-09

**Authors:** Francesco Mattei, Anna E. Hickman, Julia Uitz, Vincenzo Vellucci, Laurence Garczarek, Frédéric Partensky, Stephanie Dutkiewicz

**Affiliations:** ^1^ Sorbonne Université, CNRS, Laboratoire d’Océanographie de Villefranche, LOV Villefranche‐sur‐Mer France; ^2^ School of Ocean and Earth Science University of Southampton Southampton UK; ^3^ Sorbonne Université, CNRS, Institut de la Mer de Villefranche, IMEV Villefranche‐sur‐Mer France; ^4^ Sorbonne Université, CNRS, Adaptation and Diversity in Marine Environments, Roscoff, France; ^5^ Department of Earth Atmosphere and Planetary Sciences Massachusetts Institute of Technology Cambridge Massachusetts USA; ^6^ Center for Sustainability Science and Strategy Massachusetts Institute of Technology Cambridge Massachusetts USA

## Abstract

Phytoplankton are key components of ocean ecosystems that play a critical role in regulating Earth's climate. However, how climate‐driven changes in light availability in the ocean will affect marine phytoplankton remains poorly understood. Here, we assess the impact of climate‐induced shifts in the spectral quality of the underwater light field on the relative fitness of phytoplankton with distinct pigment traits using a global ecosystem model. We focus on *Synechococcus* pigment types, comparing light color specialists with a chromatic acclimator capable of adjusting its pigment composition. Under a high‐emission scenario, the model simulation projected an increase in the average blue‐to‐green ratio across 76% of the ocean area by the end of the 21st century, while 24% of the simulated ocean showed a shift toward greener wavelengths. Regions characterized by larger seasonal variability in blue‐to‐green ratio values appeared to be reduced due to climate‐driven spectral changes. We find that reduced variability in the ocean light field makes the chromatic acclimators' plasticity less advantageous, and this pigment type was most negatively affected. These findings highlight the potential of *Synechococcus* pigment types as functional bioindicators of ecosystem change and underscore the importance of incorporating functional diversity in global models to better predict phytoplankton responses to changing ocean conditions.

## Graphical abstract

Climate change is altering the spectral quality of the underwater light field, yet its global impact on phytoplankton pigment strategies remains unclear. Our model projections show that while the ocean is shifting toward bluer or greener states, the transitional zones characterized by variable spectral light fields are contracting. Using *Synechococcus* as a study organism, we demonstrate that this reduction in spectral variability diminishes the competitive advantage of phytoplankton with flexible pigment content (Chromatic Acclimators) relative to specialists adapted to blue or green environments. These findings highlight how resolving functional diversity enhances our ability to diagnose ecosystem changes driven by climate change.

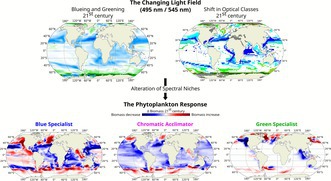

## 1 Introduction

Climate change caused by anthropogenic greenhouse gas emissions is predicted to profoundly affect the ocean, a vital component of the Earth's system that supports immense biodiversity and acts as a climate regulator (Costanza et al. 1997; Mengerink et al. 2014). Climate change is already significantly altering ocean stratification and nutrient distribution, with these effects expected to intensify and have profound consequences for marine microorganisms (Breitburg et al. 2018; Dutkiewicz, Hickman, et al. 2015; Dutkiewicz, Morris, et al. 2015; Intergovernmental Panel On Climate Change 2023). Among them, the phytoplankton community is crucial in supporting food webs, driving nutrient cycling, absorbing carbon dioxide, producing oxygen, and contributing to carbon sequestration (Duarte and Cebrián 1996; Falkowski 2012), all of which influence marine ecosystem services, including climate change mitigation (Chien et al. 2023; Fuhrman 2009; Sabine et al. 2004). By affecting the availability of fundamental resources for phytoplankton, climate change influences the dynamic feedback loop between phytoplankton communities, ocean color and underwater light field. Phytoplankton pigments absorb light at different wavebands, thereby affecting the spectral quality of underwater light. Changes in the underwater light field then shape phytoplankton community composition (Cael et al. 2023; Dutkiewicz et al. 2019; Henson et al. 2021; Kirk 1994; Kulk et al. 2020), by favoring species best adapted to the prevailing light conditions. The underwater spectral quality is also influenced by the optical properties of seawater. Pure water absorbs red light most strongly, allowing blue wavelengths to penetrate deepest in clear, oligotrophic regions, whereas higher concentrations of dissolved and particulate organic matter in coastal or turbid waters absorb blue light and shift the spectrum toward green (Kirk 1994; Stomp et al. 2007). Ocean color is already changing, with emerging patterns such as blue‐wavelength–dominated regions becoming even bluer and green‐dominated regions becoming greener (Dutkiewicz et al. 2019; Zhao et al. 2025). These shifts both influence and are influenced by the biological components of marine ecosystems. Since light is essential for photosynthesis, the inherent variability in the spectrum of light (light color) within aquatic environments has directly influenced phytoplankton evolution, driving the emergence of diverse pigments and a variety of light‐harvesting strategies (Holtrop et al. 2021; Stomp et al. 2004). Among these, chromatic acclimation enables photo‐autotrophs to reversibly adjust the relative concentrations of their photosynthetic pigments to better match the ambient light color (Sanfilippo et al. 2019; Shukla et al. 2012). This trait has evolved independently in some prokaryotes and eukaryotes (Takahashi and Mikami 2021; Voerman et al. 2023; Wolf and Blankenship 2019) to enhance light absorption and thus cell growth. Although not universal, chromatic acclimation is found across a diverse subset of taxa inhabiting freshwater, marine, and terrestrial ecosystems, and has been highlighted as a key mechanism for optimizing photosynthesis under varying light conditions (Sanfilippo et al. 2019).

While the functional and evolutionary significance of diversity in phytoplankton light‐harvesting strategies has been studied (Croce and van Amerongen 2014; Holtrop et al. 2021; Stomp et al. 2004), and regional studies have examined phytoplankton responses to altered light fields (Hintz et al. 2021; Opdal et al. 2019; Schofield et al. 2013), the global impact of climate change–driven shifts in the ocean's spectral light distribution on phytoplankton fitness remains unclear. Here, we examine how these changes influence the relative fitness of chromatic acclimators compared to other phytoplankton with more fixed pigment ratios that are adapted to specific light environments.

*Synechococcus* is an ideal candidate for investigating these questions. As the second most abundant photosynthetic organism in the ocean and a major contributor to marine primary production (Farrant et al. 2016; Flombaum et al. 2013; Paulsen et al. 2016), it also exhibits the largest pigment diversity within a single phytoplankton lineage (Olson et al. 1990; Wood et al. 1998), which is shown to be a key driver of its global distribution (Grébert et al. 2018). Its light‐harvesting antennae, known as phycobilisomes, consist of rods arranged around a central core (Sidler 1994). These rods bind various chromophorylated proteins with distinct light absorption properties, and their different configurations give rise to *Synechococcus* genotypes with distinct pigmentation, referred to as ‘pigment types’ (PTs) (Dufour et al. 2025; Grébert et al. 2022; Humily et al. 2013; Six et al. 2007). We focused on PTs containing both the green light–absorbing phycoerythrobilin (PEB) and the blue light–absorbing phycourobilin (PUB) chromophores, a combination found in the vast majority of pelagic *Synechococcus* (Grébert et al. 2018; Sunagawa et al. 2015). Some PTs are specialized in absorbing green light (green specialist, GS, exhibiting a constitutively low PUB:PEB) or blue light (blue specialist, BS, with a constitutively high PUB:PEB), while others display a variable PUB:PEB (chromatic acclimator, CA). Indeed, the CA can reversibly adjust their PUB:PEB to better match the dominant light color, thus optimizing photon absorption.

In this paper, we used a customized version of the Darwin ecosystem model (Dutkiewicz, Hickman, et al. 2015; Dutkiewicz, Morris, et al. 2015; Follett et al. 2022) that includes three *Synechococcus* PTs: GS, BS, and a CA (Mattei et al. 2025). In particular, the CA ranges from a fully green‐acclimated (PEB‐rich phycobilisome) to a fully blue‐acclimated (PUB‐rich phycobilisome) phenotype. We reveal the potential future change in these PTs in a simulation of 21st‐century ocean conditions under a high‐emission scenario (Dutkiewicz et al. 2019; Henson et al. 2021).

## 2 Materials and Methods

### 2.1 Biogeochemical Ecosystem Model

We used a biogeochemical, ecosystem, and optical numerical model (Dutkiewicz, Hickman, et al. 2015; Dutkiewicz, Morris, et al. 2015) integrated with the MIT Integrated Global System Model (IGSM). The IGSM is an integrated assessment framework linking an Earth system model of intermediate complexity to an emission model (Monier et al. 2013, 2018; Reilly et al. 2013; Sokolov et al. 2009). The Darwin model is an ideal tool for the study as it is a global scale ecosystem model embedded within a 3D global biogeochemistry and physics framework that resolves multiple plankton functional groups and size classes. Importantly, Darwin also includes spectrally resolved radiative transfer with each phytoplankton type assigned specific light absorption and scattering characteristics (Dutkiewicz, Hickman, et al. 2015; Dutkiewicz, Morris, et al. 2015; Follett et al. 2022), thereby resolving the feedbacks between phytoplankton community and the light field. Furthermore, the model has been already applied to investigate projected changes in ocean color under a high‐emission scenario, yielding robust insights into climate‐driven shifts in optical properties and community composition (e.g., Dutkiewicz et al. 2019; Henson et al. 2021; Ribalet et al. 2025). These features make the Darwin model an ideal tool for exploring the hypothesis that climate change‐driven shifts in ocean color will differentially affect the fitness of phytoplanktonic organisms exhibiting distinct light‐harvesting strategies. Here, we concisely describe the model's key features, directing readers to the cited references for additional details.

The marine biogeochemical module captures the cycling of carbon, phosphorus, nitrogen, silica, iron, and oxygen through various inorganic, living, dissolved, and particulate organic phases. The ecosystem component, based on Mattei et al. (2025), represents a range of plankton functional types, including analogues of *Prochlorococcus*, *Synechococcus*, pico‐eukaryotes of varying sizes, coccolithophores, diatoms, mixotrophs, diazotrophs, zooplankton, and heterotrophic bacteria. The plankton types' physiological rates and susceptibility to grazing are influenced by their size, with grazing determined by fixed predator–prey size ratios (Follett et al. 2022). Initial plankton functional types biomasses were set to low values to avoid artificial dominance during the spin‐up phase. The emergent global planktonic community structure arises from interactions between physical, chemical, and biological processes in the model. The optical and radiative transfer component explicitly computes the underwater light field by resolving the spectral absorption and scattering properties of seawater optically active constituents. It is based on the Ocean–Atmosphere Spectral Irradiance Model (Gregg and Casey 2009), modified to couple with the model's biogeochemical and ecosystem components (Dutkiewicz, Hickman, et al. 2015; Dutkiewicz, Morris, et al. 2015; Mattei et al. 2025). The model uses the total light absorption spectrum of the different optical components to compute light attenuation. The total light absorption spectrum includes contributions from pure seawater, detritus, colored dissolved organic matter (CDOM), and all phytoplankton groups. Each plankton type is characterized by a specific total and photosynthetic absorption spectrum, which determines light attenuation and the light available for photosynthesis, respectively. The model resolves the photosynthetically active spectral range (400–700 nm) at 5‐nm intervals, allowing accurate representation of narrow pigment absorption peaks such as those of PUB (495 nm) and PEB (545 nm) (Mattei et al. 2025). This fine spectral resolution enables realistic simulation of blue‐to‐green light gradients and their feedback on phytoplankton community structure (Mattei et al. 2025). The implementation of *Synechococcus* PTs in the Darwin model was grounded in experimental data and culture‐based data (Mattei et al. 2025). Three PTs were represented: BS, GS, and CA. These types share identical physiological and ecological parameters (e.g., maximum growth rate, nutrient affinity, and temperature response) and differ only in their wavelength‐specific absorption spectra. The BS and GS were parameterized as PUB‐ and PEB‐dominant types (PUB/PEB ≈1.4 and 0.4, respectively), while the CA was modeled with six intermediate acclimation states (CA_1_–CA_6_) spanning the physiological transition between blue‐ and green‐acclimated conditions (PUB/PEB from ~0.67 to ~1.4). Intermediate spectra were generated by linear interpolation between experimentally measured end members, reproducing the gradual pigment adjustments observed in cultured strains (Dufour et al. 2024; Humily et al. 2013; Mattei et al. 2025). The duration of blue‐green acclimation in *Synechococcus* is influenced by irradiance, ranging from about 6 days under low light conditions (~20 μmol photons m^−2^ s^−1^) to approximately 3 days when exposed to higher light levels (~75 μmol photons m^−2^ s^−1^), consistent with laboratory observations (Dufour et al. 2024; Humily et al. 2013; Mattei et al. 2025). The model was validated against the Tara Oceans metagenomic dataset, the most comprehensive survey of *Synechococcus* pigment diversity to date, showing correct identification of the dominant PT in 66% of sampling stations and a Matthews correlation coefficient of 0.44 (Mattei et al. 2025). This quantitative comparison demonstrates the model's ability to capture broad spatial and temporal dynamics of global ocean patterns. The model was parameterized from targeted laboratory experiments to test ecological outcomes of a well‐resolved physiological process, without tuning parameters to fit field data. The agreement between the model's PT distribution and global patterns established in the literature further underscores the robustness of this experimentally grounded approach. We refer to Mattei et al. (2025) paper, Supporting Information and associated Zenodo data repository (https://zenodo.org/records/14283389) for full details about the PTs parameterization and model validation.

The model simulation has a 50‐year spin‐up phase, allowing the phytoplankton community and upper‐ocean biogeochemistry to reach quasi‐equilibrium. This was followed by a 250‐year simulation spanning 1860 to 2110. The model has a horizontal resolution of 2° × 2.5° (latitude × longitude) with 22 vertical layers, ranging from 10 m at the surface to 500 m at depth. The marine biogeochemical tracers are advected and mixed using the MIT General Circulation Model (Marshall et al. 1997). The broader IGSM framework also incorporates representations of atmospheric dynamics, physics, chemistry, terrestrial processes, and a global carbon cycle.

Our analysis focuses on a high emission scenario (Monier et al. 2013) resulting in over 1000 ppmv CO_2_‐equivalent atmospheric concentrations and globally almost 4°C increase in sea surface temperature by the end of the century. Details and evaluation of the earth system model and projections are detailed in Monier et al. (2013). For the simulations here, the ecosystem was driven by physical fields from the Earth system model under pre‐industrial conditions during the spin‐up. Subsequently, the 1860–2110 simulation period was conducted. The Darwin model's detailed spectral resolution and ability to simulate dynamic interactions among plankton analogues under varying physio‐chemical conditions made it an essential tool for investigating the effects of climate change on light‐harvesting strategies and ecosystem structuring. A control run with atmospheric greenhouse gas concentrations held at pre‐industrial levels was conducted and showed no significant trends in the ecological or biogeochemical components of the model.

### 2.2 Data Management and Statistical Analyses

Model results of *Synechococcus* biomass, acclimation index, the ratio of irradiance at 495 nm (blue light) to 545 nm (green light), referred to as B/G, and chlorophyll *a* are provided as 10‐year averages to account for natural temporal variability and isolate the differences caused by climate change. Specifically, the values for the year 2000 represent an average over the period 1995–2005, while those for the year 2100 are based on the 2095–2105 period. All maps presented in this study follow this approach, ensuring that the results reflect long‐term trends rather than short‐term (interannual) fluctuations. This method provides a more robust comparison between present and future conditions, highlighting the impact of climate change on the analyzed variable. Similarly, the annual variance of the B/G for the years 2000 and 2100 was computed by first calculating the annual variance for each year of the 10‐year time window and then averaging the variance of the 10 years.

The acclimation index, which quantifies the extent to which the chromatic acclimation trait is utilized, is derived from the number of acclimation states (Figure S2–S4, white numbers) coexisting in the water column throughout the year. To calculate this, the total number of CA acclimation states present at each depth and month was summed (Figure S2–S4, red numbers on the right side of the figures between the main plot and the colorbar). This variable was then normalized between 0 and 1 based on the minimum and maximum values from the 10‐year average at the beginning of the century. The same procedure was applied to the end‐of‐century data, using the normalization scaling range established from the early‐century values to ensure comparability. While the absolute value of the index is partly dependent on the chosen number of discrete acclimation states included in the model and the temporal resolution of the output used for analysis (in this case, six acclimation states and monthly intervals), the acclimation index serves as a practical and interpretable indicator of the variability in the number of acclimation states coexisting across depth and time. Although its magnitude might shift slightly if calculated over different time intervals, the acclimation index provides a meaningful measure of how much chromatic acclimation was exploited within a given light environment.

To assess the relationship between the acclimation index and the biomass of CA, we employed two statistical measures: the Matthews Correlation Coefficient (Matthews 1975) and Spearman's Rank Correlation (Spearman 1904). The Matthews Correlation Coefficient evaluates the agreement in sign between the two variables, indicating whether they are both positive or negative. Spearman's Rank Correlation, a non‐parametric test, measures the strength and direction of a monotonic relationship between the variables. Unlike Pearson's correlation, which assumes a linear relationship, Spearman's correlation captures both linear and non‐linear monotonic relationships, providing a more flexible assessment of how the acclimation index and CA biomass vary together.

## 3 Results

The Darwin high‐emissions simulation predicts a global decrease in the annual average phytoplankton biomass of approximately 4% by the end of the century (Figure S1). Alongside this overall decline, climate change is projected to significantly alter the distribution of planktonic biomass, leading to an increase at higher latitudes and a decrease at lower latitudes. This latitudinal pattern is consistent across several modeling studies (e.g., Kwiatkowski et al. 2020; Tittensor et al. 2021).

### 3.1 Ocean Light Field Alteration Induced by Climate Change

The model predicted significant long‐term trends in ocean color associated with changes in the spectral composition of available light at the global scale (Dutkiewicz et al. 2019). Decadal‐scale changes in ocean colour are already being observed in satellite data and related to changes in phytoplankton (Wernand et al. 2013), supporting the premise of long‐term change in these interrelated properties. To investigate the effects of climate change on *Synechococcus* light‐harvesting strategies, we focused on the B/G. Since the wavelengths at 495 nm and 545 nm correspond to the absorption peaks of PUB and PEB respectively, the B/G is directly related to the light‐harvesting capabilities of these PTs. This ratio does not only help identify favourable light conditions for different PTs but also reflects the combined influence of physical, chemical, and biological feedback in marine ecosystems. As such, it serves as a valuable proxy for analyzing complex dynamics, such as the expansion of gyres (Polovina et al. 2008; Signorini et al. 2015) induced by climate change.

**FIGURE 1:**
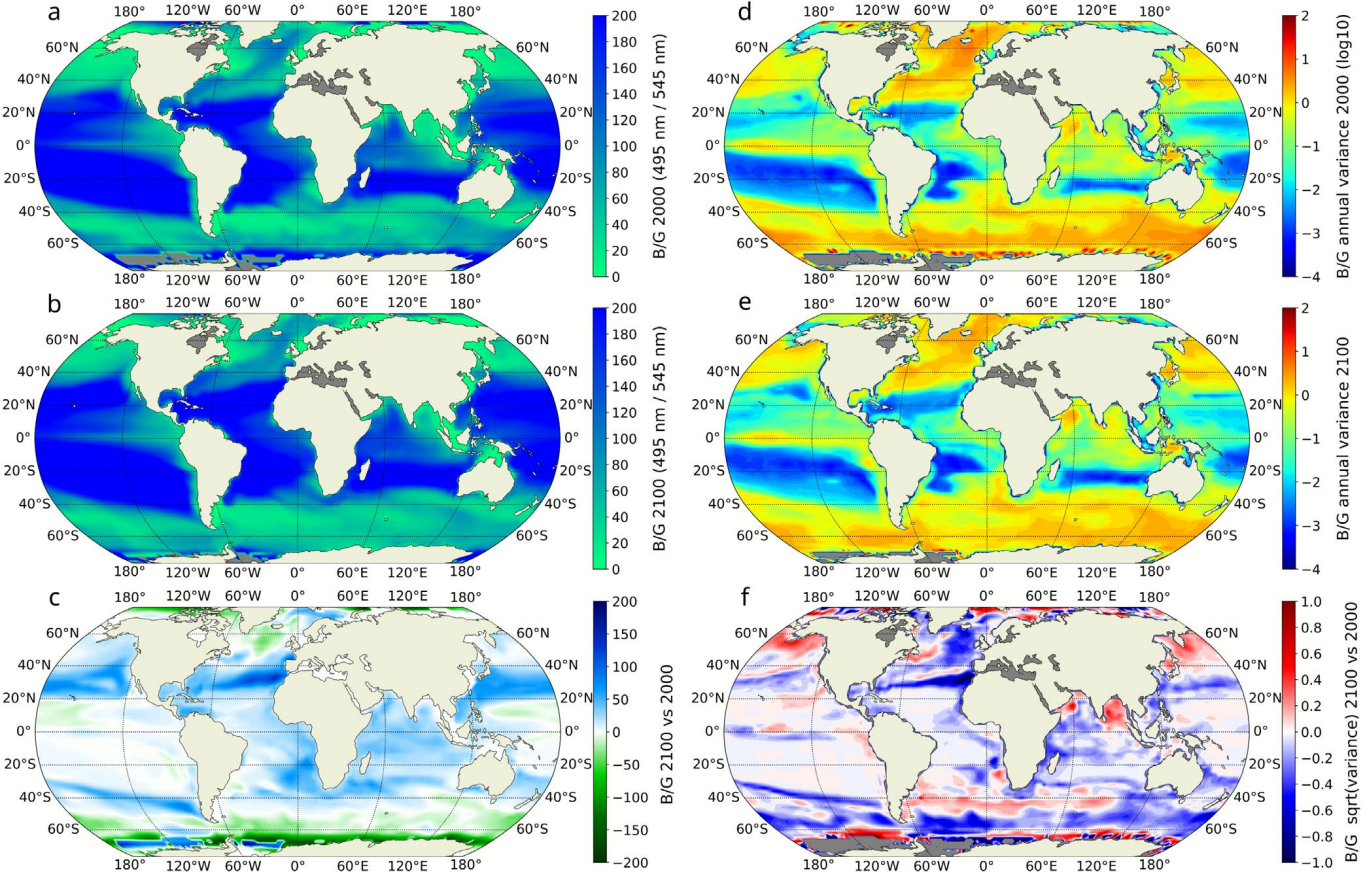
Global average maps of the blue‐to‐green light ratio (B/G, 495 nm / 545 nm). (a, b) Average proportion of blue (495 nm) and green (545 nm) wavelengths in the simulated ocean within the first 200 m for the beginning (2000) and end (2100) of the century, respectively. (c) Difference map of the average B/G between years 2100 and 2000. Blue shades indicate regions where blue wavelengths increased, while green shades indicate regions where green wavelengths increased by 2100. (d, e) Maps of the B/G annual variance for 2000 and 2100, representing the seasonal variability of the ratio. (f) Difference map of the B/G annual variance, highlighting areas where seasonal variability is projected to increase (positive values) or decrease (negative values) by the end of the century. All the maps represent a 10‐year average around the nominal year (i.e., 1995–2005 for the year 2000 and 2095–2105 for the year 2100).

The model simulation projected an increase in the average B/G across 76% of the ocean area by the end of the 21st century (Figure 1a–c), while 24% of the simulated ocean area showed a shift toward greener wavelengths. The shift toward bluer wavelengths is most pronounced in the centers and along the edges of subtropical gyres, a pattern particularly evident in the Atlantic and North Pacific Oceans (Figure 1c) where biomass decreased. The simulation shows a decrease in the B/G by 2100, primarily in regions already associated with low ratio values, such as higher latitudes due to increased phytoplankton biomass.

**FIGURE 2:**
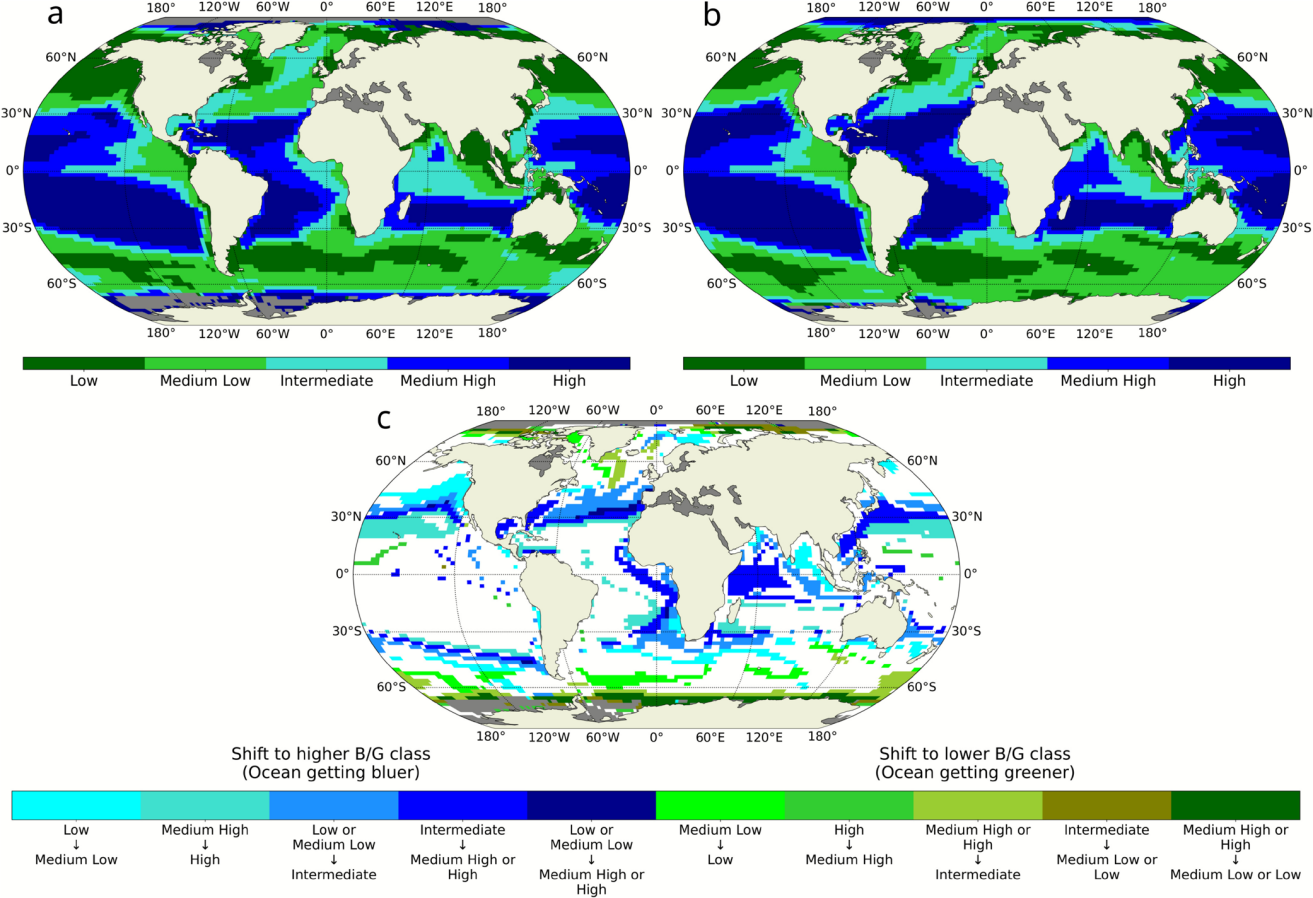
Global classification of the blue‐to‐green light ratio (B/G). The B/G annual average is clustered into five magnitude classes using the Fisher‐Jenks algorithm to identify natural breaks at the beginning (a) and end (b) of the 21st century. (c) Global maps of changes in B/G classes from 2000 to 2100. Colors indicate whether a given region remained in the same class (white), or shifted toward a bluer (blue shades) or greener class (green shades) by the end of the century.

To further investigate changes in the ocean light field, we classified B/G values into five magnitude classes (from low to high) using the Fisher‐Jenks algorithm, which groups similar values while maximizing differences between classes (Figure 2a,b). Our analysis revealed that 35% of the ocean area experienced a shift in B/G classification, with most changes reflecting a transition toward a bluer ocean (higher B/G class). Specifically, 27% of the ocean area shifted to a higher B/G class, while only 8% transitioned to a lower B/G class. Overall, the global changes led to a net decline in the extent of the low and medium‐low B/G classes, which contracted by 2% and 1%, respectively (Figure 2 and Table S1). Although part of this decline resulted from transitions into the intermediate class, which migrated to higher latitudes and coastal waters, the total area classified as intermediate still decreased by 3%. In contrast, the medium‐high and high B/G classes were the only ones to expand, increasing by 1.4% and 5%, respectively. Notably, much of this increase originated from regions initially classified as intermediate (turquoise pixels, Figure 2c).

Despite these shifts, 65% of the ocean area retained its original optical class (Table S2). The largest share of this stable area belonged to the very high B/G class (20%), followed by the low (13%), medium‐low (13%), and medium‐high (11%) classes. The intermediate class represented only 7% of the unchanged area, highlighting its greater vulnerability to climate‐driven reclassification.

Given our particular focus on the CA, we also analyzed changes in the variability of B/G, specifically the annual variance at the beginning (Figure 1d) and end of the century (Figure 1e), thereby highlighting regions that will experience either an increase or a decrease in seasonal variability. The results show a decrease of B/G annual variance in 64% of the ocean (Figure 1f) suggesting a loss in annual spectral light variability. Areas where the B/G is projected to increase by 2100 can be categorized into three main groups: (i) Blue light‐dominated waters (high B/G) that are expected to become even bluer (Figure S2). Such areas are typically associated with low B/G annual variance, like the oligotrophic gyres (Figure 1 where nutrient scarcity limits phytoplankton production), resulting in an enhanced and more stable dominance of blue wavelengths throughout the year. (ii) Intermediate B/G areas with higher seasonality compared to the gyres (Figure S3) that are projected to become bluer. These areas experience a reduction in B/G seasonality, as seen in the temperate North Atlantic. (iii) low B/G waters that are projected to experience an increase in blue wavelengths (Figure S4; Figure 1a,b). These areas are associated with amplified seasonal variability in B/G (Figure 1d,e). Examples include the Gulf of Alaska in the North Pacific, the Bay of Bengal in the Indian Ocean, and the boundaries between the Southern Ocean and the Atlantic and Indian Oceans.

### 3.2 Climate Change Impacts on *Synechococcus* Distribution and Pigment Diversity

**FIGURE 3:**
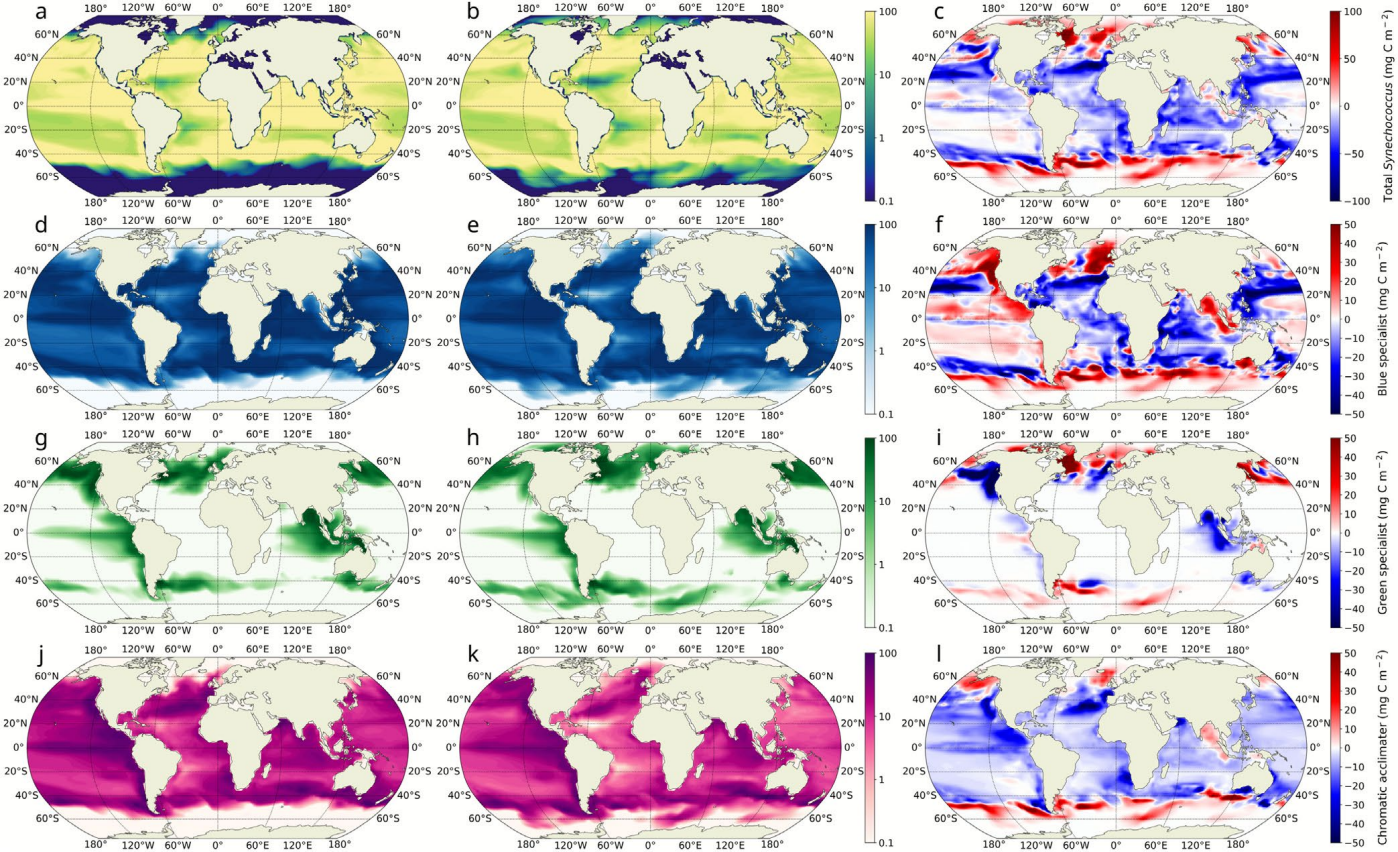
Global average biomass distribution of the whole *Synechococcus* population and of the different pigment types. a, d, g, and j show the annual average biomass distribution of *Synechococcus* integrated over 200 m, BS, GS, and CA at the beginning of the 21st century. b, e, h, and k represent the annual average biomass distribution of the same groups at the end of the 21st century. c, f, i, and l illustrate the annual average biomass differences between years 2100 and 2000 for total *Synechococcus*, BS, GS, and CA, respectively. Red shades indicate an increase in biomass for a given group, while blue shades represent a decrease. The maps represent a 10‐year average around the nominal year (i.e., 1995–2005 for the year 2000 and 2095–2105 for the year 2100).

The The general response of *Synechococcus* to a high‐emission scenario aligns with the overall pattern projected for total phytoplankton biomass, with a reduction at lower latitudes and an increase at higher latitudes (Figure 3a–c), resulting in a reduction overall. This poleward expansion encompasses increased biomass within already colonized areas and the extension of the *Synechococcus* realized niche in the simulation. Incorporating three distinct *Synechococcus* PTs into the Darwin model enabled a detailed analysis of different light‐harvesting strategies and their effectiveness in coping with climate change‐driven shifts in ocean light field. Our simulation revealed that, by the end of the 21st century, all PTs experienced an overall biomass decline while simultaneously expanding their realized niches toward higher latitudes compared to the beginning of the century. However, the responses of individual PTs showed distinct patterns. The CA, despite its flexible phenotype, was the least well‐suited to the future ocean under climate change. It experienced a biomass loss of ~23%, and its contribution to total *Synechococcus* biomass declined from ~22% to ~18% (Figure 3j–l). The BS experienced a biomass loss of ~4%, yet its contribution to the total *Synechococcus* biomass increased from ~68% to ~71% by 2100 (Figure 3d–f). The poleward expansion of this PT's realized niche was more pronounced in the Southern Ocean than in the Arctic, and it was the only PT to exhibit a biomass increase in the Pacific Ocean. The GS, the least abundant PT, experienced a minor global biomass loss of < 1% but showed a slight increase in its share of total *Synechococcus* biomass from ~10% to ~11%. While these global changes are small, local variations were more pronounced (Figure 3g–i). The realized niche of the GS showed a larger expansion in the Arctic compared to the Southern Ocean. Overall, the CA was the worst affected by future change, both in terms of overall biomass decrease and in the realized niche. The poleward expansion of the CA's realized niche was less pronounced than both of the specialists'.

### 3.3 Climate Change Reduces the Advantage of Chromatic Acclimation

**FIGURE 4:**
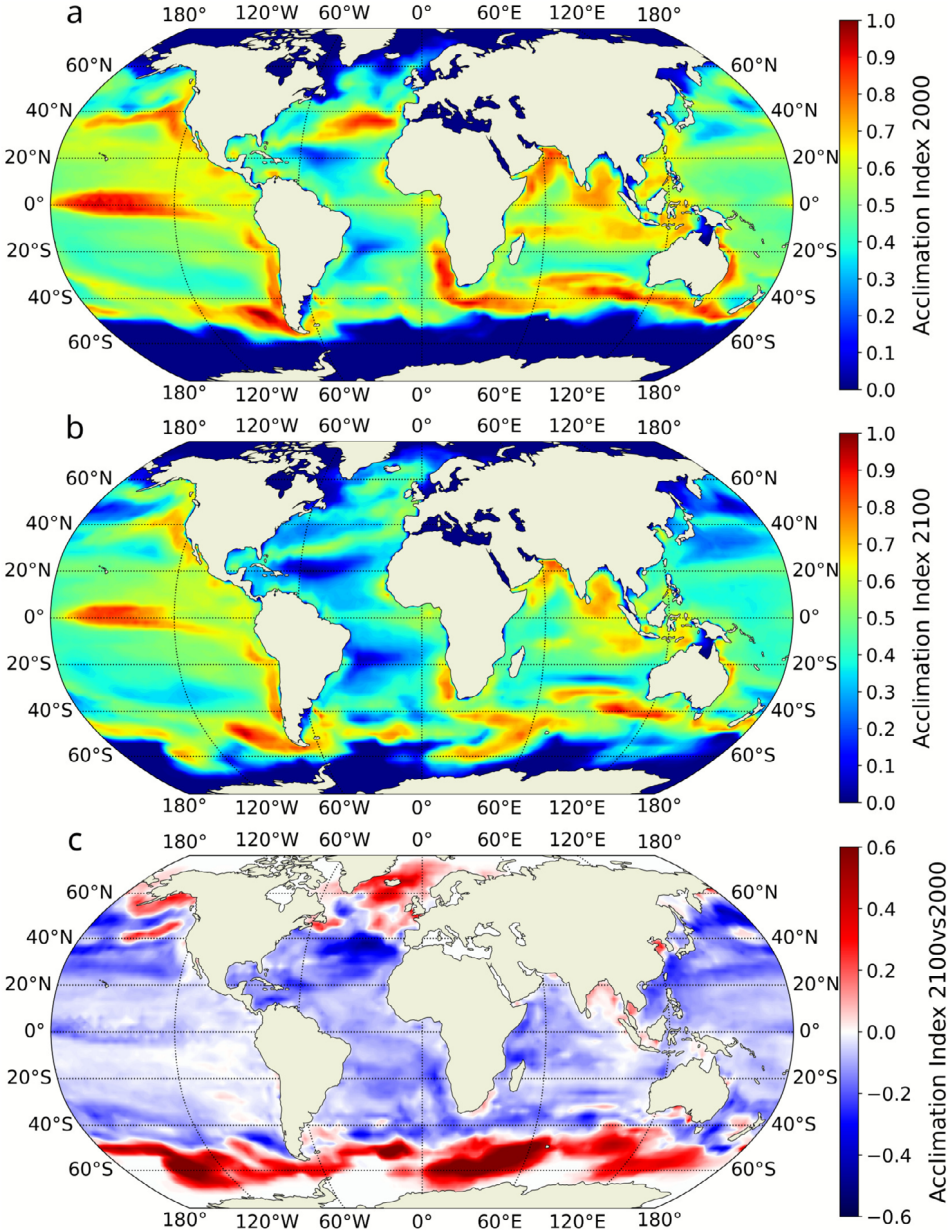
Global distribution of the acclimation index. (a, b) Annual average acclimation index for 2000 and 2100, respectively. (c) Difference in the acclimation index between years 2100 and 2000, highlighting regions where the exploitation of the chromatic acclimation process is projected to increase (red shades) or decrease (blue shades) by the end of the 21st century. The maps represent a 10‐year average around the nominal year (i.e., 1995–2005 for the year 2000 and 2095–2105 for the year 2100).

To further explore the disadvantage of CA in the simulation, we analysed the global distribution and the change of the ‘acclimation index’, a proxy indicating the extent to which the chromatic acclimation strategy was exploited in the simulated ocean. High index values indicate the coexistence of multiple acclimation states in the model, driven by a highly variable vertical and temporal light field (Mattei et al. 2025), while low values suggest fewer dominant acclimation states, reflecting a more stable light environment (see methods for further details). This index showed a decrease in 77.5% of the simulated ocean area by the end of the century (Figure 4a–c). The decrease of the acclimation index was most pronounced in the oligotrophic gyres of the North Atlantic Ocean and the transitional zones between the gyres and coastal or temperate waters. A similar decline, albeit less marked, was observed in the Pacific and Indian Oceans (Figure 4c). Conversely, the acclimation index increased notably across much of the Southern Ocean, as well as in the Arctic Ocean and in specific regions of the Pacific and Indian Oceans, including the Gulf of Alaska and the Bay of Bengal.

**FIGURE 5:**
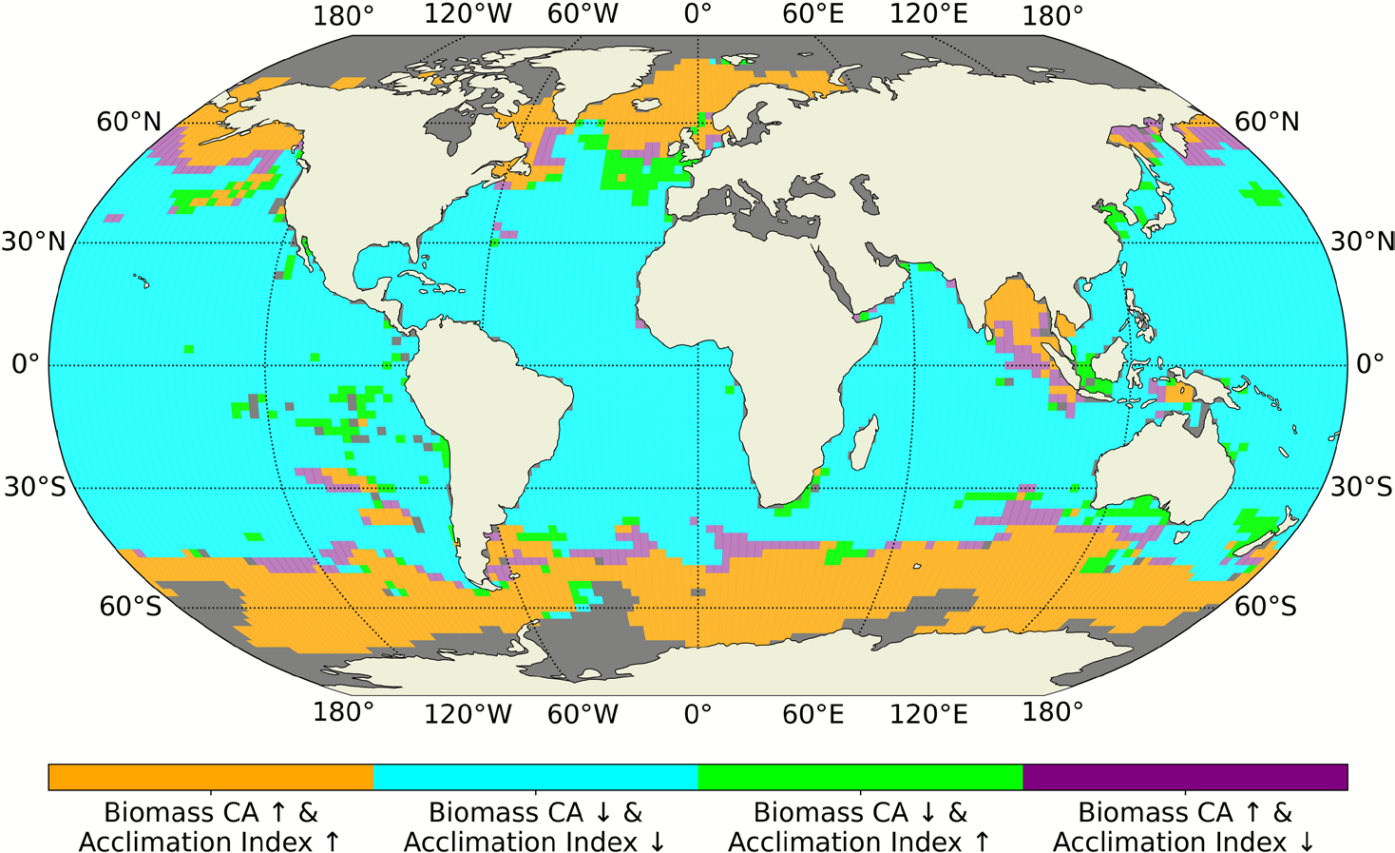
Spatial relationship between chromatic acclimator biomass change and acclimation index change. Orange pixels indicate regions where CA biomass and the acclimation index will increase by the end of the 21st century. Cyan pixels represent areas where both variables decrease between years 2000 and 2100. The overall agreement in the direction of change between CA biomass and the acclimation index is reflected by the combined sum of orange and cyan pixels, accounting for 91.7% of the simulated ocean area with a Matthews correlation coefficient of 0.8 and a Spearman's rank correlation of 0.67. Green and purple pixels indicate regions where the two variables exhibit opposite directional change, representing ~8.3% of the simulated ocean.

To test the hypothesis that the decline in CA biomass might result from a reduced advantage conferred by chromatic acclimation, we examined the relationship between the change in CA biomass (Figure 3l) and the change in the acclimation index (Figure 4c) from the beginning to the end of the 21st century (Figure 5). The two variables showed consistent directional changes in 91.7% of the simulated ocean area, with a Matthews correlation coefficient (Matthews 1975) of 0.8 and a Spearman's rank correlation (Spearman 1904) of 0.67.

## 4 Discussion

Understanding how climate change alters the underwater light field is essential for predicting phytoplankton responses, as spectral niches strongly influence community structure and ecosystem functioning (Dutkiewicz et al. 2019; Hintz et al. 2021; Holtrop et al. 2021; Stomp et al. 2007). Here, we examined how simulated climate change reshapes ocean optical properties and showed, in particular, the decreased competitiveness of cells able to adjust their pigment content via chromatic acclimation compared to specialists with fixed pigmentation under a high‐emission scenario. The contrasts in our model organisms, the *Synechococcus* PTs, reveal for the first time the complex, region‐specific effects of climate change on the underwater light spectrum and phytoplankton composition. The regional heterogeneity of response represents a robust ecological signal of community turnover in a warming ocean and aligns with expectations of spatially uneven shifts in phytoplankton composition under climate change (Bopp et al. 2013; Dutkiewicz et al. 2013; Henson et al. 2021; Kwiatkowski et al. 2020; Lotze et al. 2019; Tittensor et al. 2021). Consistent with previous modelling studies, our simulation predicts a latitudinal redistribution of phytoplankton biomass driven by enhanced stratification, altered circulation, changes to nutrient limitation (Kwiatkowski et al. 2020; Moore et al. 2018; Tittensor et al. 2021) and light environment. Declining biomass in much of the subtropical gyres, expanding areal extent of the subtropical gyres, and increasing biomass in some high latitude regions modify the available spectral and ecological niches (Davies and Smyth 2025). The physical–biogeochemical shifts propagate into substantial changes in the underwater light field.

Our analysis revealed a global shift in the underwater light spectrum toward bluer wavelengths over much of the lower latitudes, driven by a reduction in phytoplankton biomass (Figure S1). This trend aligns with previous studies on climate‐induced optical changes in the ocean (Dutkiewicz et al. 2019; Irwin and Oliver 2009; Leonelli et al. 2022; Polovina et al. 2008). Classification of the B/G ratio showed that approximately one‐quarter of the global ocean is projected to become bluer. In regions already dominated by blue light, most notably the oligotrophic subtropical gyres, this shift is driven by intensified upper‐ocean stratification, which reduces nutrient supply and suppresses phytoplankton biomass (Moore et al. 2018; Kwiatkowski et al. 2020). Biomass changes lead to higher B/G and reduced seasonal variability of the ratio. Conversely, around a tenth of the ocean became greener, primarily at high latitudes, including in the Arctic. This was driven by warming‐enhanced growth rates and increased light availability (due to sea‐ice melt and higher stratification) which increased phytoplankton biomass and lowered the B/G ratio.

As subtropical gyres expand, the intermediate B/G regions, typically found at gyre edges and characterised by high temporal spectral variability, were pushed poleward and toward coastal zones. However, there was also an overall contraction of these transitional light environments. This reduction reflected the combined effects of poleward expansion of blue oligotrophic gyres and the intensification of greener, biomass‐rich waters at high latitudes, while coastlines act as fixed boundaries that compress the remaining intermediate regions between expanding gyres and the continents. The corresponding loss of spectral variability narrows the range of light environments available to photosynthetic organisms, especially those adapted to variable light colour.

Climate change is projected to expand the overall distribution of *Synechococcus*, particularly into temperate and high‐latitude regions (Figure 3c) where enhanced stratification and warming reduce nutrient levels and disadvantage larger, low‐affinity phytoplankton groups (Dutkiewicz et al. 2013; Dutkiewicz et al. 2024). In most regions that it had originally existed, though, the biomass of *Synechococcus* decreases in the projected future ocean, due to lower nutrient availability. However, *Synechococcus* PTs respond differently to these environmental changes because of their distinct light‐harvesting strategies (Figure 3f,i,l). Where green wavelengths intensify, especially at high latitudes, GS remains the most efficient competitor and maintains its dominance over both BS and CA. Where blue wavelengths strengthen, such as in the expanding oligotrophic gyres or in intermediate regions shifting toward higher B/G (Figure 2c, turquoise pixels), BS gains a competitive advantage over CA. Reduced seasonal variability in B/G diminishes the advantage of pigment flexibility, leading to lower chromatic acclimation index values (Figure 4) and enabling BS to outcompete CA. This mechanism explains the pronounced losses of CA biomass in low‐latitude oligotrophic regions, where decreasing variability eroded its niche. Conversely, the subtropical gyre expansion leads to an increase in blue light together with higher seasonal variability in B/G in regions that were previously dominated by green light during the growing season. In these areas, GS loses its competitive edge, allowing both BS and CA to expand. The resulting increase in the acclimation index indicates that CA is increasingly able to exploit light‐color niches that were previously unavailable, contributing to localized biomass gains despite broader declines elsewhere. However, the contraction of the intermediate zone is particularly disadvantageous to CA, whose realized niche is intrinsically tied to environments with strong temporal variability in light color (Mattei et al. 2025).

The overarching trend, blue regions becoming bluer and green regions becoming greener, marks a fundamental restructuring of the ocean's spectral irradiance landscape. Shifts toward greener higher latitudes and bluer oligotrophic regions are already likely underway (Zhao et al. 2025). This may appear to be in contradiction to the findings of Cael et al. (2023), who suggested a greening over some regions of the ocean, but we caution that that study focused on multi‐waveband changes. We believe that the trends in the blue may not yet have been significant in the 20 years that Cael et al. (2023) considered. Here, using a model, we show that the greening‐green and blueing‐blue are also accompanied by a contraction of transitional zones characterized by variable spectral light fields, an overlooked but critical effect of climate change. Reduction in transitional zones as well as increased stratification led to a decline in the regional extent to which the chromatic acclimation trait can be exploited, showing that this strategy becomes less advantageous under future climate conditions. The reduced spectral heterogeneity projected under climate changes likely diminishes the competitive advantage of chromatic acclimation relative to color specialists adapted to narrower spectral niches. Such a reduction in spectral variability may have already been captured in previous climate simulations, but it likely went unnoticed because those models lacked the functional diversity necessary to translate subtle optical shifts into ecological signals. By resolving distinct PTs, our approach exposes how these physical–optical trends propagate into biological consequences, revealing an overlooked dimension of ocean change.

These spectral shifts have important ecological implications. Stratification and the contraction of the transitional zones alter the competition in favor of specialists (BS, GS) adapted to stable light environments, while generalist strategies, such as those represented by the CA, which perform better under variable light conditions, decline. Because generalist strategies act as ecological buffers (Cardinale et al. 2012), their decline could therefore reduce ecosystem resistance, making communities more vulnerable to disturbances. In contrast, specialists often lack the flexibility to adapt to abrupt environmental changes. A reduced representation of generalist strategies could thus erode functional diversity, increasing the risk of regime shifts and cascading impacts throughout the marine food web (Duarte 2000; Worm et al. 2006). This study shows how biological diversity serves as a lens through which subtle environmental shifts can be detected and interpreted. The distinct responses of the three *Synechococcus* PTs to projected light‐field changes demonstrate that functional diversity can reveal complex ecosystem adjustments that would remain overlooked in bulk biomass estimates. In this sense, diversity acts not only as an outcome of environmental variability but also as a bioindicator of ecosystem change. Incorporating such trait‐based diversity into ecosystem models therefore enhances their diagnostic and predictive power, allowing them to capture feedbacks and vulnerabilities that simplified representations of phytoplankton communities would overlook. Ultimately, this approach improves our ability to anticipate and manage climate‐driven transformations in ocean ecosystems.

## Author Contributions

**Francesco Mattei:** conceptualization, methodology, software, validation, formal analysis, investigation, data curation, writing – original draft, writing – review and editing, visualization. **Anna E. Hickman:** conceptualization, writing – original draft, writing – review and editing, methodology, software, investigation, resources, supervision, project administration. **Julia Uitz:** conceptualization, writing – original draft, writing – review and editing, resources, supervision, project administration, funding acquisition. **Vincenzo Vellucci:** conceptualization, writing – original draft, writing – review and editing, funding acquisition. **Laurence Garczarek:** conceptualization, writing – original draft, writing – review and editing, resources, supervision, project administration, funding acquisition. **Frédéric Partensky:** conceptualization, writing – original draft, writing – review and editing, resources, supervision, project administration, funding acquisition. **Stephanie Dutkiewicz:** conceptualization, methodology, software, investigation, resources, writing – original draft, writing – review and editing, supervision, project administration, funding acquisition.

## Funding

This work was supported by the French “Agence Nationale de la Recherche” programs EFFICACY (ANR‐19‐ce02‐0019) and TaxCy (ANR‐23‐ce2‐0007) and the Simons Collaboration on Computational Biogeochemical Modeling of Marine Ecosystem (CBIOMES) (549931 to S.D.).

## Conflicts of Interest

The authors declare no conflicts of interest.

## Supporting information


**Data S1:** gcb70671‐sup‐0001‐Supinfo.docx.

## Data Availability

All data and code necessary to ensure the reproducibility of the results are available in a Zenodo repository https://zenodo.org/records/18165233; DOI: https://doi.org/10.5281/zenodo.18165232.
