# Supplementary material for: Phytoplankton With Flexible Pigment Content Disadvantaged by Projected Future Decrease in Variability of the Ocean Light Spectrum
Source: Glob Chang Biol. 2026 Jan 9;32(1):e70671. doi: 10.1111/gcb.70671 (PMC12789851; doi:10.1111/gcb.70671)
Supplement: Supplementary file 1 — Data S1: gcb70671‐sup‐0001‐Supinfo.docx. [file GCB-32-e70671-s001.docx]

**Supplemental Tables and Figures**

**Table S1: Global ocean area distribution across B/G classes in 2000 and 2100.** The table reports the percentage of the ocean area clustered into each B/G magnitude class at the beginning (2000) and end (2100) of the century, along with changes in area associated with each class. Positive values indicate an increase in the extent of a given class, while negative values represent a decline.


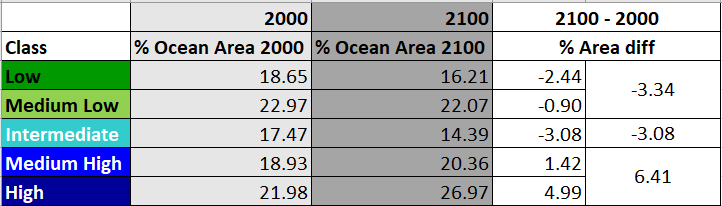


**Table S2. Changes in blue-to-green ratio (B/G) classes from 2000 to 2100.** The table presents the percentage of the global ocean area that remained in the same B/G class or transitioned to a different class. Rows indicate whether a region remained stable ("Same Class"), shifted toward a bluer ocean ("Bluer"), or shifted toward a greener ocean ("Greener"). The colors correspond to those in Figure 5d.


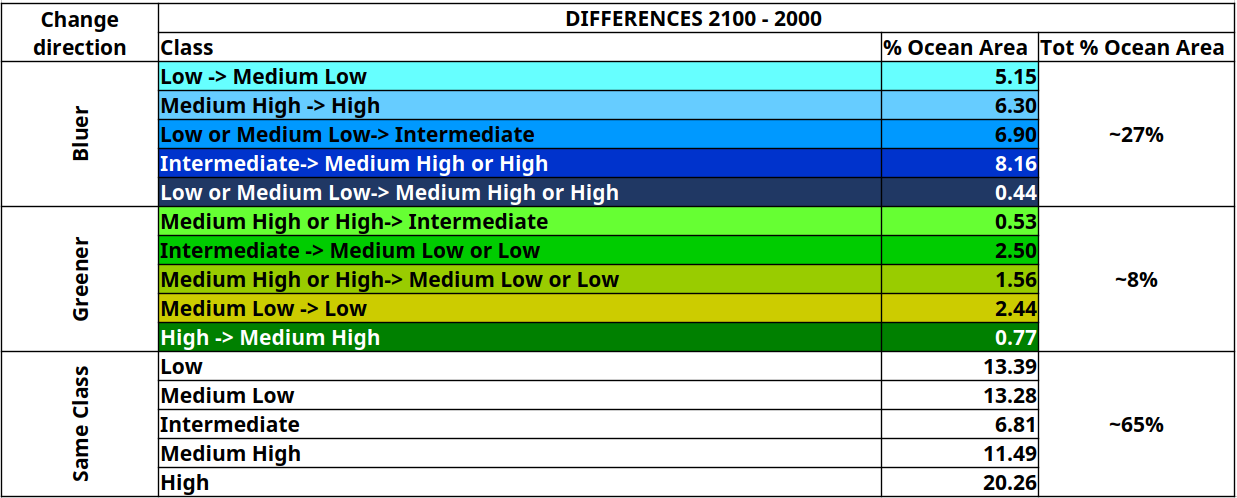


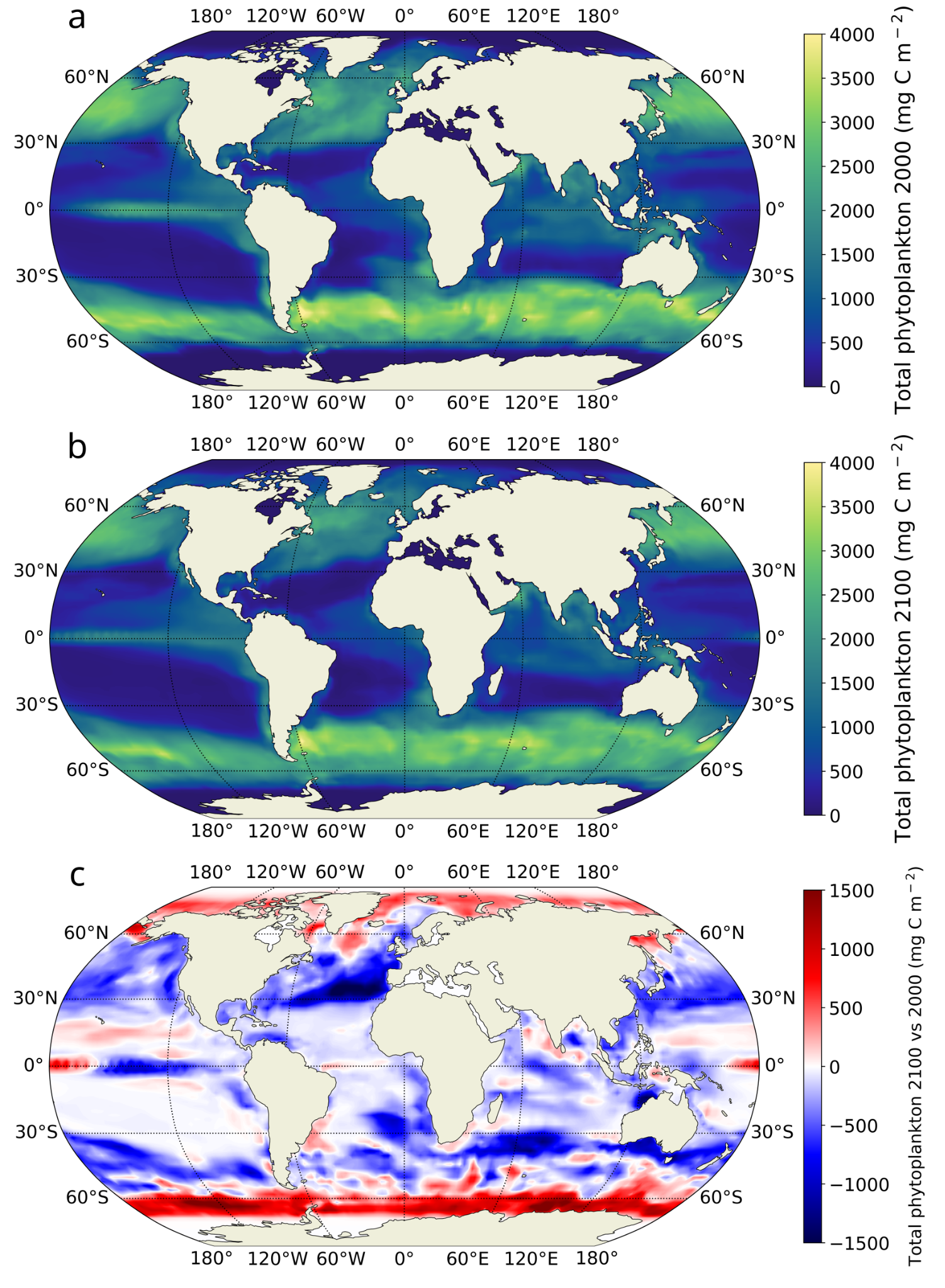


**Fig. S1: Global average phytoplankton biomass distribution**. Global distribution of the annual average phytoplankton biomass integrated over 200 m at the beginning (**a**) and the end of the 21st century (**b**). (**c**) Difference in biomass between years 2100 and 2000, with red shades indicating an increase and blue shades indicating a decrease in total phytoplankton biomass.


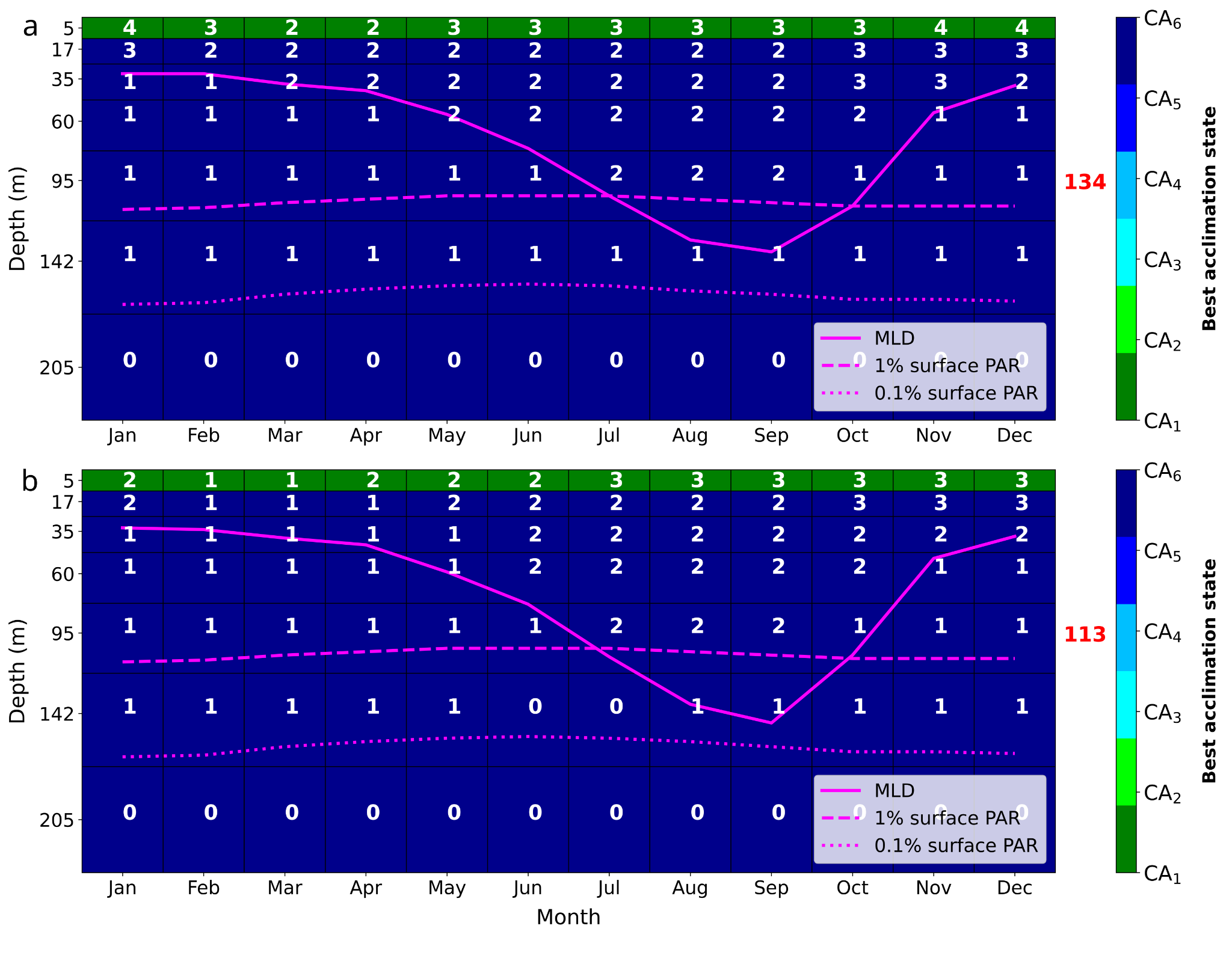


**Figure S2. Vertical profiles of the number of acclimation states coexisting in the water column at an example oceanic gyre location (30.5°S, 31.5°E)**. This example illustrates a region already dominated by blue wavelengths at the beginning of the 21 st century (**a**), where this dominance intensifies by the end of the century (**b**) due to climate change. The white numbers indicate the number of coexisting acclimation states in each depth bin. The red number between the plot and the color bar represents the total number of acclimation states coexisting throughout the water column over the year, which is used to compute the acclimation index. From the beginning (**a**) to the end of the century (**b**), reduced variability in the light field leads to a lower number of coexisting acclimation states. The background color of each depth bin indicates the acclimation state most efficient at harvesting light (best acclimation state), providing a proxy for the available light quality at each depth.


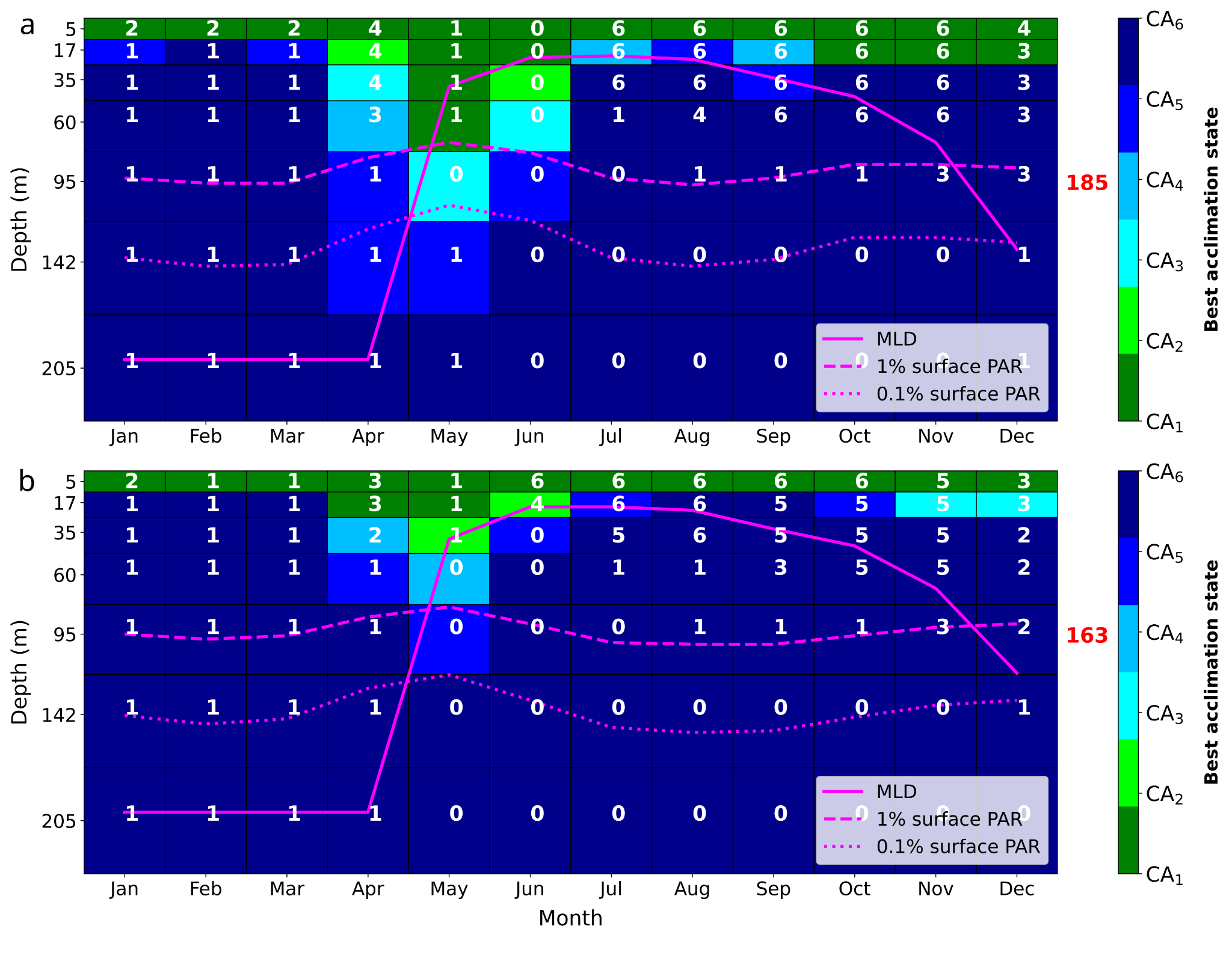


**Figure S3. Vertical profiles of the number of acclimation states coexisting in the water column at an example temperate location (44.5°N, 30.5°E)**. This example illustrates a region with greater seasonal light field variability at the beginning of the 21st century (a) compared to the end of the century (b), where climate change-induced increases in blue wavelengths lead to a loss of seasonal variability. The white numbers indicate the number of coexisting acclimation states in each depth bin. The red number between the plot and the color bar represents the total number of acclimation states coexisting throughout the water column over the year, which is used to compute the acclimation index. The background color of each depth bin indicates the acclimation state most efficient at harvesting light (best acclimation state), providing a proxy for the available light quality at each depth. The reduction in light field variability is reflected by a decrease in the number of coexisting acclimation states and a decline in the diversity of best acclimation states.


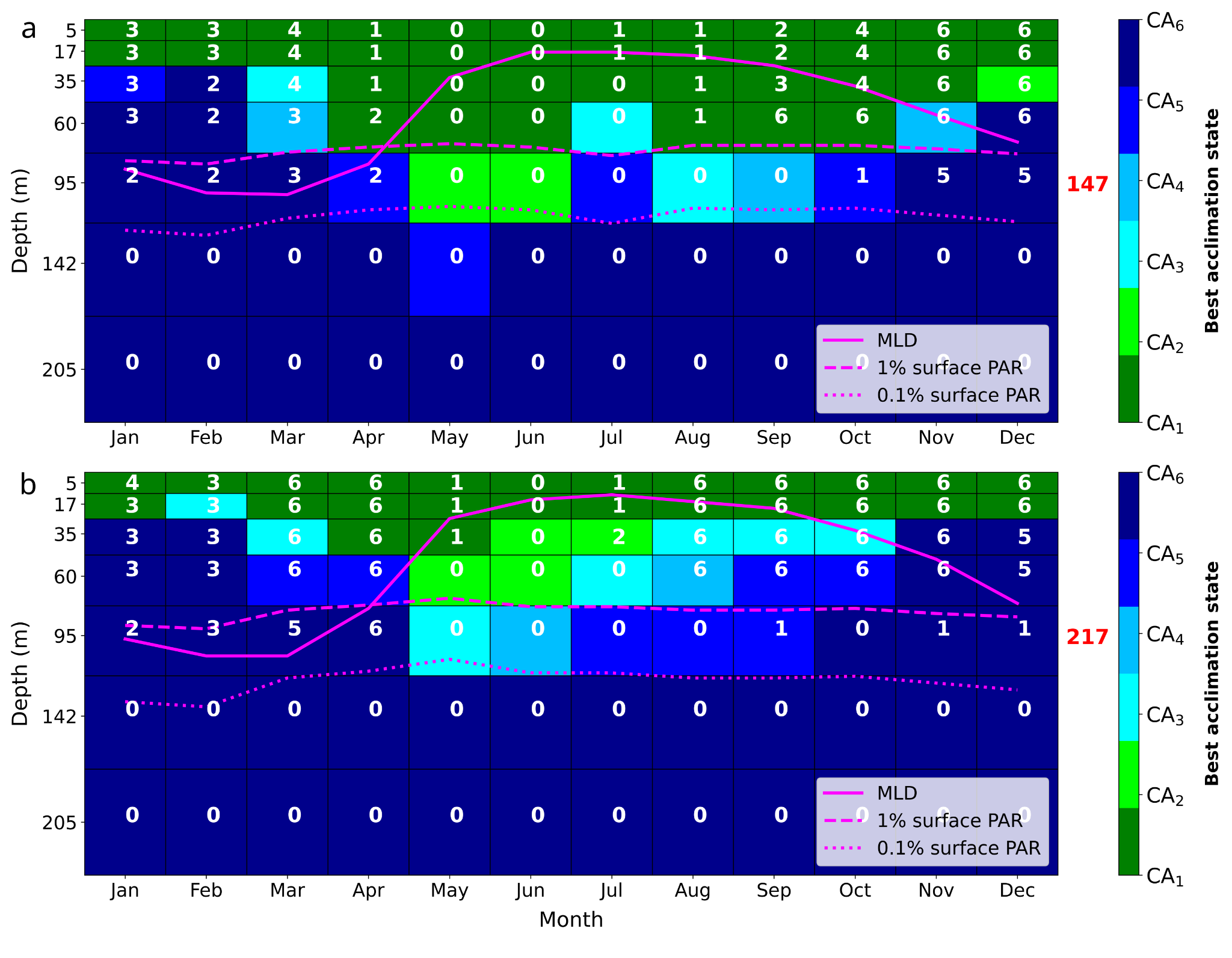


**Figure S4. Vertical profiles of the number of acclimation states coexisting in the water column at an example location dominated by green wavelengths** **(latitude: 53° N, longitude: 153° E).** In this example, the green specialist dominated year-round due to the prevalence of green wavelengths in the light field (**a**). By the end of the 21st century (**b**), an increase in blue wavelengths led to greater light field variability, allowing the blue specialist and chromatic acclimator to compete with the green specialist. The white numbers indicate the number of coexisting acclimation states in each depth bin. The red number between the plot and the color bar represents the total number of acclimation states coexisting in the water column throughout the year, which is used to compute the acclimation index. From the beginning (**a**) to the end of the century (**b**), the increased variability in the light field results in a higher number of coexisting acclimation states.
